# Prevention of Arterial Stiffening by Using Low-Dose Atorvastatin in Diabetes Is Associated with Decreased Malondialdehyde

**DOI:** 10.1371/journal.pone.0090471

**Published:** 2014-03-04

**Authors:** Chih-Hsien Wang, Ru-Wen Chang, Ya-Hui Ko, Pi-Ru Tsai, Shoei-Shen Wang, Yih-Sharng Chen, Wen-Je Ko, Chun-Yi Chang, Tai-Horng Young, Kuo-Chu Chang

**Affiliations:** 1 Department of Surgery, National Taiwan University Hospital, Taipei, Taiwan; 2 Department of Surgery, National Taiwan University Hospital, Hsin-Chu Branch, Hsin-Chu, Taiwan; 3 Department of Surgery and Traumatology, National Taiwan University Hospital, Taipei, Taiwan; 4 Department of Physiology, College of Medicine, National Taiwan University, Taipei, Taiwan; 5 Department of Emergency Medicine, National Taiwan University Hospital, Taipei, Taiwan; 6 Institute of Biomedical Engineering, College of Medicine and Engineering, National Taiwan University, Taipei, Taiwan; The Ohio State Unversity, United States of America

## Abstract

**Introduction:**

Without affecting the lipid profile, a low-dose treatment with atorvastatin contributes to the reduction of oxidative stress, inflammation, and adverse cardiovascular events in diabetes. In this study, we investigated whether low-dose atorvastatin exerts any beneficial effect on vascular dynamics in streptozotocin (STZ)-induced diabetes in male Wistar rats.

**Methods:**

Diabetes was induced using a single tail-vein injection of STZ at 55 mg kg^−1^. The diabetic rats were treated daily with atorvastatin (10 mg kg^−1^ by oral gavage) for 6 weeks. They were also compared with untreated age-matched diabetic controls. Arterial wave reflection was derived using the impulse response function of the filtered aortic input impedance spectra. A thiobarbituric acid reactive substances measurement was used to estimate the malondialdehyde content.

**Results:**

The high plasma level of total cholesterol in the diabetic rats did not change in response to this low-dose treatment with atorvastatin. Atorvastatin resulted in a significant increase of 15.4% in wave transit time and a decrease of 33.5% in wave reflection factor, suggesting that atorvastatin may attenuate the diabetes-induced deterioration in systolic loads imposed on the heart. This was in parallel with its lowering of malondialdehyde content in plasma and aortic walls in diabetes. Atorvastatin therapy also prevented the diabetes-related cardiac hypertrophy, as evidenced by the diminished ratio of left ventricular weight to body weight.

**Conclusion:**

These findings indicate that low-dose atorvastatin might protect diabetic vasculature against diabetes-associated deterioration in aorta stiffness and cardiac hypertrophy, possibly through its decrease of lipid oxidation-derived malondialdehyde.

## Introduction

An increase in oxidative chemical modifications of tissue proteins has been implicated in the pathogenesis of diabetes mellitus [1], [2]. High glucose levels and free fatty acids (FFAs) in diabetes can act in concert to stimulate the production of reactive oxygen species (ROS) [3], [4]. Persistent hyperglycemia also results in the formation of advanced glycation end products (AGEs), which leads to increased oxidative stress [5]. Malondialdehyde (MDA) is a highly toxic by-product formed in part by lipid peroxidation derived free radicals [6], [7]. Slatter et al. [8], [9] have shown that MDA can react with long-lived proteins, such as glycated collagen to form MDA-collagen cross-links that not only stabilize the collagen but render it susceptible to further glycation. Thus, the pathogenic cross-linking of collagen through MDA may affect tissue remodeling and result in loss of elasticity, contributing to the development of certain physical changes of the vasculature.

Statins, the competitive inhibitors of 3-hydroxy-3-methylglutaryl coenzyme A (HMG-CoA) reductase, have been shown to reduce cardiovascular events and mortality in diabetics [10], [11]. The protective effects of statins on cardiovascular system are mainly explained by its lipid-lowering property. On the other hand, statins have many other effects, including anti-inflammation [12], anti-oxidative stress [13], and improving endothelial function [14]. These cholesterol-independent effects of atorvastatin (Ator), that is, pleiotropic effects, could contribute at least in part to the reduction in cardiovascular events. Because oxidative stress generation participates in the formation of lipid oxidation-derived MDA, inhibition of the MDA formation is supposed to be a novel molecular target of Ator.

Without affecting the lipid profile, a low-dose treatment with Ator contributes to the reduction of oxidative stress, inflammation, and adverse cardiovascular events in diabetes [15]. For this study, we investigated whether a low-dose Ator had any benefit on vascular dynamics in streptozotocin (STZ)-induced diabetes in rats. We also evaluated its abilities to reduce the highly toxic MDA content in diabetes. The physical properties of the arterial system were assessed using the aortic input impedance that was the frequency relationship between pulsatile pressure and flow signals measured in the ascending aorta [16]–[18]. Plasma levels of FFA and total cholesterol, as well as plasma and aorta levels of MDA were also detected. We further determined the effects of Ator on the AGE-derived modification of aortic collagen in diabetes using the immunohistochemical staining and western blotting techniques.

## Materials and Methods

### Animals and catheterization

**Figure 1:**
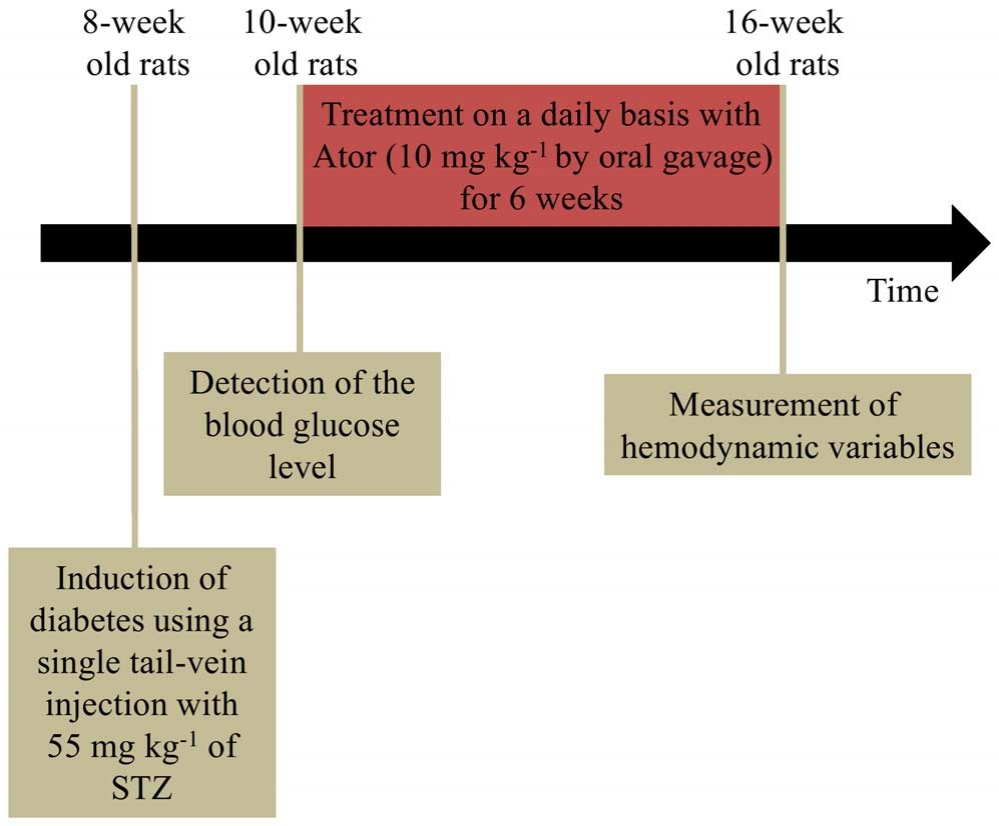
Scheme for the description of the time point the rats were induced diabetes mellitus and treated with Ator. Ator, atorvastatin; STZ, streptozotocin.

Two-month-old male Wistar rats were randomly divided into 4 groups, as follows: (1) normal controls (NC) (*n* = 14); (2) NC+Ator (*n* = 14); (3) STZ-induced diabetic rats (DM) (*n* = 14); (4) DM+Ator (*n* = 14). Diabetes was induced in animals by a single tail-vein injection with 55 mg kg^−1^ of STZ in a 0.1 *M* citrate buffer (pH 4.5) (Sigma Chemical Co., St. Louis, MO, USA). The blood glucose level was determined using a SURESTEP Test Strip (Lifescan Inc., Milpitas, CA, USA) to confirm the development of hyperglycemia. Two weeks after the development of hyperglycemia, the diabetic rats were treated on a daily basis with Ator (10 mg kg^−1^ by oral gavage) for 6 weeks (Fig. 1). They were also compared with untreated age-matched diabetic controls. Animals were allowed free access to Purina Chow and water with a 12-hour light/dark cycle. The experiments were conducted according to the *Guide for the Care and Use of Laboratory Animals*, and our study protocol was approved by the Animal Care and Use Committee of National Taiwan University.

General surgical procedures and measurement of hemodynamic variables in anesthetized rats have previously been described [17]. Animals were anesthetized using intraperitoneal sodium pentobarbital (50 mg kg^−1^), placed on a heating pad, intubated, and ventilated with a rodent respirator (Model 131; New England Medical Instruments, Medway, MA, USA). The chest was opened through the second intercostal space on the right side. An electromagnetic flow probe (model 100 series, internal circumference 8 mm, Carolina Medical Electronics, King, NC, USA) was positioned around the ascending aorta to measure the pulsatile aortic flow. A high-fidelity pressure catheter (model SPC 320, size 2F; Millar Instruments, Houston, TX, USA) was used to measure the pulsatile aortic pressure through the isolated carotid artery on the right side. The electrocardiogram (ECG) of lead II was recorded with a Gould ECG/Biotech amplifier (Gould Electronics, Cleveland, OH, USA). The selective pressure and flow signals from 5 to 10 beats were averaged in the time domain, using the peak R-wave of ECG as a fiducial point. Timing asynchronicity between the pressure and flow signals (caused by the spatial distance between the flow probe and the proximal aortic pressure transducer) was corrected using a time-domain approach, in which the foot of the pressure waveform was realigned with that of the flow [19]. The resulting pressure and flow signals were subjected to further vascular impedance analysis.

At the end of the experiment, each rat was sacrificed to obtain the weight of the left ventricle (LV). The ratio of the LV weight (LVW) to body weight (BW) was used as an indicator of the degree of cardiac hypertrophy.

### Aortic Input Impedance Spectra

The aortic input impedance spectra (*Z_i_*) were obtained from the ratio of the ascending aortic pressure harmonics to the corresponding flow harmonics by using a standard Fourier series expansion technique [16]–[18]. The total peripheral resistance of systemic circulation (*R_p_*) was calculated as the mean aortic pressure divided by the mean aortic flow. The aortic characteristic impedance (*Z_c_*) was calculated by averaging the high-frequency moduli of the aortic input impedance data points (4th–10th harmonics). Considering *Z_c_*, we calculated the systemic arterial compliance *C_m_* at mean aortic pressure *P_m_*, as follows:
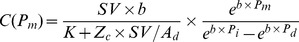
where *SV* was the stroke volume; *K* was the ratio of the total area under the aortic pressure curve to the diastolic area (*A_d_*); *b* was the coefficient in the pressure-volume relation (−0.0131±0.009 in the aortic arch); *P_i_* was the pressure at the time of incisura; and *P_d_* was the end-diastolic pressure [17], [20].

**Figure 2:**
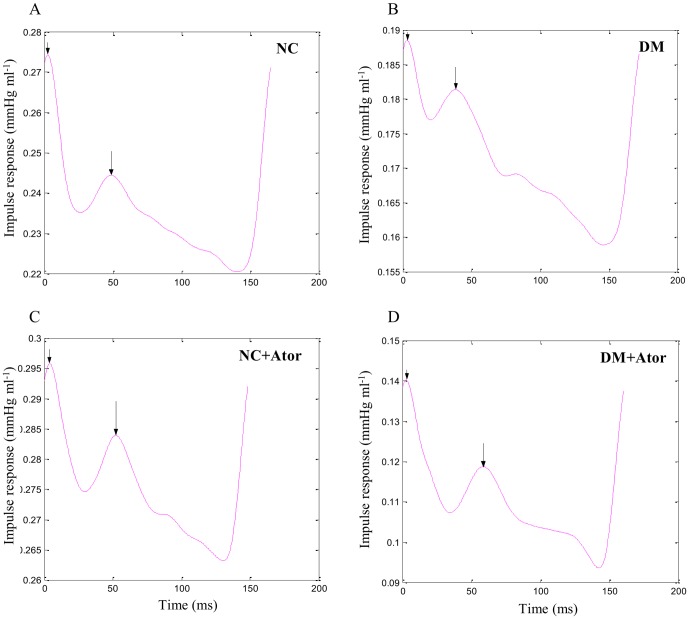
Impulse response function curve from a DM+Ator rat (D) compared with that of an untreated diabetic rat (B). The long arrow shows the discrete reflection peak from the body circulation, and the short arrow demonstrates the initial peak as a reference. Half of the time difference between the appearance of the reflected peak and the initial peak approximates the wave transit time in the lower body circulation. The low-dose treatment with Ator attenuates the diabetes-induced abnormality in timing of the pulse wave reflection, as evidenced by the increase in wave transit time. NC, normal controls; DM, STZ-diabetic rats; Ator, atorvastatin; STZ, streptozotocin.

The wave transit time (τ) was calculated according to the impulse response of the filtered *Z_i_* (Fig. 2). This calculation was accomplished using the inverse transformation of *Z_i_* after multiplication of the first 12 harmonics by using a Dolph-Chebychev weighting function with order 24 [21]. The long arrow in Fig. 2 indicates the discrete reflection peak from the body circulation and the short arrow demonstrates the initial peak as a reference. Half of the time difference between the long and short arrows approximated the arterial τ in the lower body circulation [22]. The time-domain reflection factor (*R_f_*) was derived from the amplitude ratio of the backward-to-forward peak pressure waves, using the method proposed by Westerhof et al. [23]. Therefore, both the wave transit time and the wave reflection factor characterized the wave reflection phenomenon in the vasculature.

### Measurement of Plasma Levels of FFAs and Total Cholesterol

At the end of catheterization, blood samples from the rats studied were collected using cardiac puncture, and were subsequently centrifuged at 1600 g at 4°C for 10 minutes to determine the non-esterified fatty acid concentrations. The plasma levels of FFA and total cholesterol were determined using their respective enzymatic kits (Cayman, Ann Arbor, MI, USA and Biovision, Mountain View, CA, USA) [24], [25].

### Detection of High Sensitivity C-Reactive Protein (hsCRP) Using Enzyme-Linked Immunosorbent Assay (ELISA)

Quantification of plasma levels of hsCRP (ALPCO, NH) was performed using commercially available ELISA kits in strict accordance with the manufacturer's instructions.

### Estimate of MDA Contents in Plasma and Aortas by Using a Thiobarbituric Acid (TBA) Assay

Although MDA is not the only physiological molecule that can react with TBA [6], [26], the TBA assay is the most frequently used assay for MDA. Based on this method, the results were TBA reactive substances (TBARS) instead of MDA. Therefore, TBARS were used as an estimate of MDA.

At the end of catheterization, the rat heart was perfused with phosphate-buffered saline (PBS). The thoracic aortas were dissected and quickly washed with ice-cold PBS, and immediately frozen using liquid nitrogen. The frozen tissues were stored in a −80°C till analysis. The tissues were homogenized in a RIPA buffer (Sigma Chemical Co) with a 1% protease inhibitor cocktail (Sigma Chemical Co), and centrifuged at 1600 g at 4°C for 10 min to obtain supernatants for the MDA measurement. The MDA contents in plasma and aortas were estimated by TBARS using a commercial kit (Cayman) [27]. The protein concentrations of aortas were assayed using the Bradford method (DC Protein Assay, Bio-Rad, Hercules, CA, USA) [28].

### Immunohistochemical Staining for AGEs

Rat aortic rings were fixed in 4% (w/v) formalin and embedded in paraffin. The sections were then reacted overnight at 4°C with primary polyclonal antibodies, namely rabbit anti-AGE IgG (ab23722) (1∶10000; Abcam, Cambridge, UK), all in TBS containing 0.1% Triton X-100. The sections were then incubated with secondary antibody Fab' fragments for 1 h at room temperature using Histofine Simple Stain MAX PO (M) (anti-mouse/rabbit) (Nichirei Biosciences Inc., Tokyo, Japan) following the manufacturer's instructions. Finally, sections were visualized with 3, 3′-diaminobenzidine tetrahydrochloride hydrate (DAB; Dako Cytomation; K3466). Hematoxylin nuclear staining (Sigma-aldrich, Missouri, USA) was also applied. All tissue sections were mounted on gelatin-coated slides (Dako Cytomation) and embedded with Permount (Fisher; SP-15-100). As for quantification of immunostaining, 10 different fields on the cross section of the aorta were randomly photographed for each animal. Digital micrographs of 1280×1024 pixels were taken at 400× agnification. ImageScope Viewer^R^ (Aperio Inc, Vista, CA, USA) was used to quantify the strength of AGE expression. By using the Positive Pixel Count algorithm, ImageScope Viewer^R^ was able to label and count the pixels expressing different intensity. The positivity, or the percentage of AGE staining, could therefore be calculated by the following formula:

Positivity (%)  =  Total number of positive pixels divided by total number of pixels

### Western Blot Analysis for AGEs and Pentosidine

Rat aortic tissues were pulverized at −80 by using a pestle and mortar and resuspended in RIPA buffer (1% Nonidet P-40, 0.5% sodium deoxycholate, 0.1% SDS and 1% protease inhibitors cocktail). The homogenates were centrifuged at 12,000×g, 4°C for 15 min and supernatant was collected. The total protein concentration of the supernatant was determined by using the Bradford reagent (Sigma-aldrich, Missouri, USA). Proteins (50 µg each lane) were separated by 8% SDS-polyacrylamide gels electrophoresis (Mini-Protein III, Bio-Rad) and electrotransferred onto 0.2 µm PVDF membrane (Bio-Rad, Hercules, CA). The membranes were blocked overnight with 5% (w/v) nonfat milk in PBST buffer (PBS buffer with 0.05% (w/v) Tween 20) and incubated overnight with primary antibodies: rabbit polyclonal anti-AGEs antibody (ab23722) (1∶200; Abcam, Cambridge, UK) or mouse monoclonal anti-pentosidine (1∶100; Pen-12, Trans Genic Inc, Tokyo, Japan) in the rat aorta whole cell lysate. The membranes were exposed to horseradish peroxidase-conjugated anti-rabbit (or anti-mouse) IgG secondary antibody (1∶2000; Abcam, Cambridge, UK) for 1 hour, and immunoreactivity was visualized using an ECL detection system (PerkinElmer, MA, USA). Autoradiographic films were volume-integrated within a linear range of exposure using a scanning densitometer. Relative quantity was obtained by normalizing the density of target protein against that of β-actin.

### Statistics

The results were expressed as means ± s.e. Because cardiac output is significantly related to body shape, this variable was normalized to BW when a comparison was made between the DM and the age-matched controls. Other hemodynamic variables derived from blood flow were also normalized to BW to detect the effects of Ator on these parameters with or without diabetes. Two-way ANOVA was used to assess the hemodynamic and metabolic effects of Ator in the DM. A simple effect analysis was performed when a significant interaction between diabetes and Ator occurred. Differences among means within levels of a factor were determined using Tukey's honestly significant difference (HSD) method. Statistical significance was defined at *P*<0.05.

## Results

Table 1 shows the effects of Ator on blood glucose level, BW, LVW, plasma hsCRP, and the aortic pressure profile in the DM. The high glucose level in the DM did not change in response to Ator. After exposure to Ator, the DM demonstrated a significant increase in BW, but did not differ in LVW compared with the untreated diabetic controls. The diabetes-related increase in the ratio of LVW to BW was attenuated by treatment of the DM with Ator. Although there was a trend toward decreasing plasma hsCRP levels, the Ator therapy with this low dose of 10 mg kg^−1^ did not significantly diminish this pro-inflammatory cytokine in rats with insulin deficiency. Ator also produced no significant changes in the aortic pressure profile in the DM. Variables were not affected by administration of Ator to the NC, with the exception of BW and LVW.

**Table 1:** Effects of Ator on blood glucose level, body weight, left ventricular weight, plasma CRP, and aortic pressure profile in the STZ-diabetic rats.

Variable	NC (*n* = 14)	NC+Ator (*n* = 14)	DM (*n* = 14)	DM+Ator (*n* = 14)
BS (mg dl^−1^)	95.1±3.24	96.6±3.03	488.6±13.8†	467.6±15.1
BW (g)	464.3±10.5	423.2±8.6*	308.2±3.7†	336.8±8.9‡
LVW (g)	0.85±0.03	0.77±0.02*	0.73±0.02†	0.72±0.01
LVW/BW (mg g^−1^)	1.84±0.04	1.83±0.03	2.37±0.06†	2.15±0.03‡
hsCRP (ng ml^−1^)	131.3±1.1	130.9±2.9	328.1±3.1†	316.9±2.9
*P_s_* (mmHg)	117.1±2.5	120.0±3.5	111.2±3.7	115.9±2.7
*P_d_* (mmHg)	92.4±2.7	95.7±3.7	85.7±3.8	88.2±2.6
*P_m_* (mmHg)	105.7±2.8	108.9±3.6	99.5±3.6	103.7±2.4

All values are expressed as means ± s.e. BS, blood sugar; BW, body weight; LVW, left ventricular weight; LVW/BW, ratio of the LVW to BW; hsCRP, high sensitivity C-reactive protein; *P_s_*, systolic aortic pressure; *P_d_*, diastolic aortic pressure; *P_m_*, mean aortic pressure; NC, normal controls; NC+Ator, NC treated with Ator; DM, STZ-diabetic rats; DM+Ator, DM treated with Ator; Ator, atorvastatin.

*P<0.05 when the NC+Ator was compared with the NC;

† *P*<0.05 when the DM was compared with the NC;

‡ *P*<0.05 when the DM+Ator was compared with the DM.

Figure 1 was the scheme that described the time point the rats were induced diabetes mellitus and treated with Ator. Figure 2 exemplifies the impulse response function curve from a DM+Ator rat compared with that of an untreated diabetic rat. Treatment of the DM with Ator induced a clear increase in wave transit time (time difference between the appearance of the reflected peak and the initial peak), suggesting that Ator attenuated the diabetes-induced abnormality in the timing of the pulse wave reflection.

**Figure 3:**
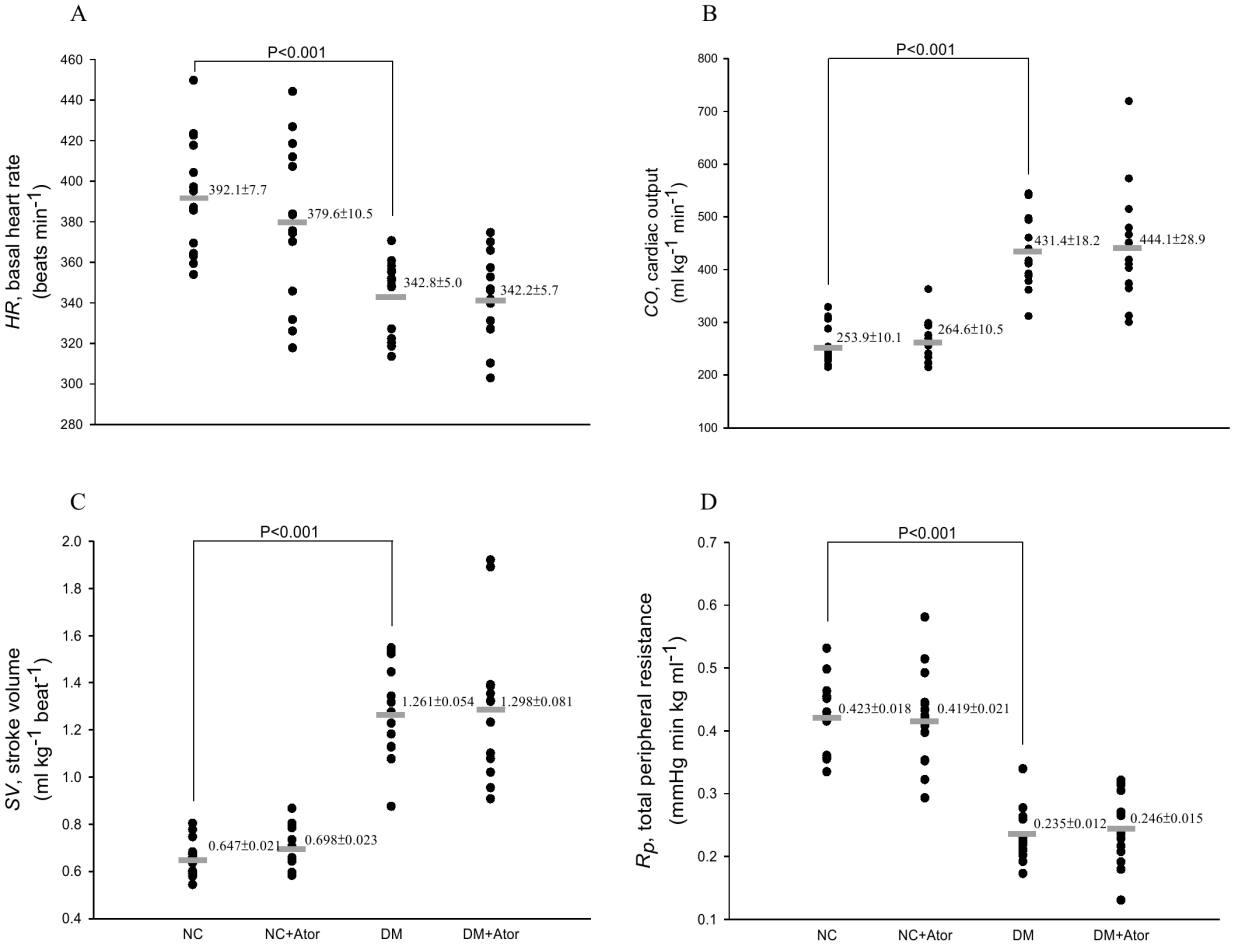
Effects of diabetes and Ator on the static hemodynamic loads such as basal heart rate (*HR* in A), cardiac output (*CO* in B), stroke volume (*SV* in C), and total peripheral resistance (*R_p_* in D). Data are expressed as means ± s.e. NC, normal controls; DM, STZ-diabetic rats; Ator, atorvastatin; STZ, streptozotocin. (*n* = 14 in each group).

Figure 3 illustrates the effects of diabetes and Ator on basal heart rate (*HR* in A), cardiac output (*CO* in B), stroke volume (*SV* in C), and total peripheral resistance (*R_p_* in D). The diabetes-related decline in *HR* was not prevented by the administration of Ator to the rats treated with STZ. A significant increase in both *CO* and *SV* and a decrease in *R_p_* with diabetes were also not affected in response to the Ator treatment. In addition, the Ator therapy exerted no effects on those static hemodynamic variables in the NC.

**Figure 4:**
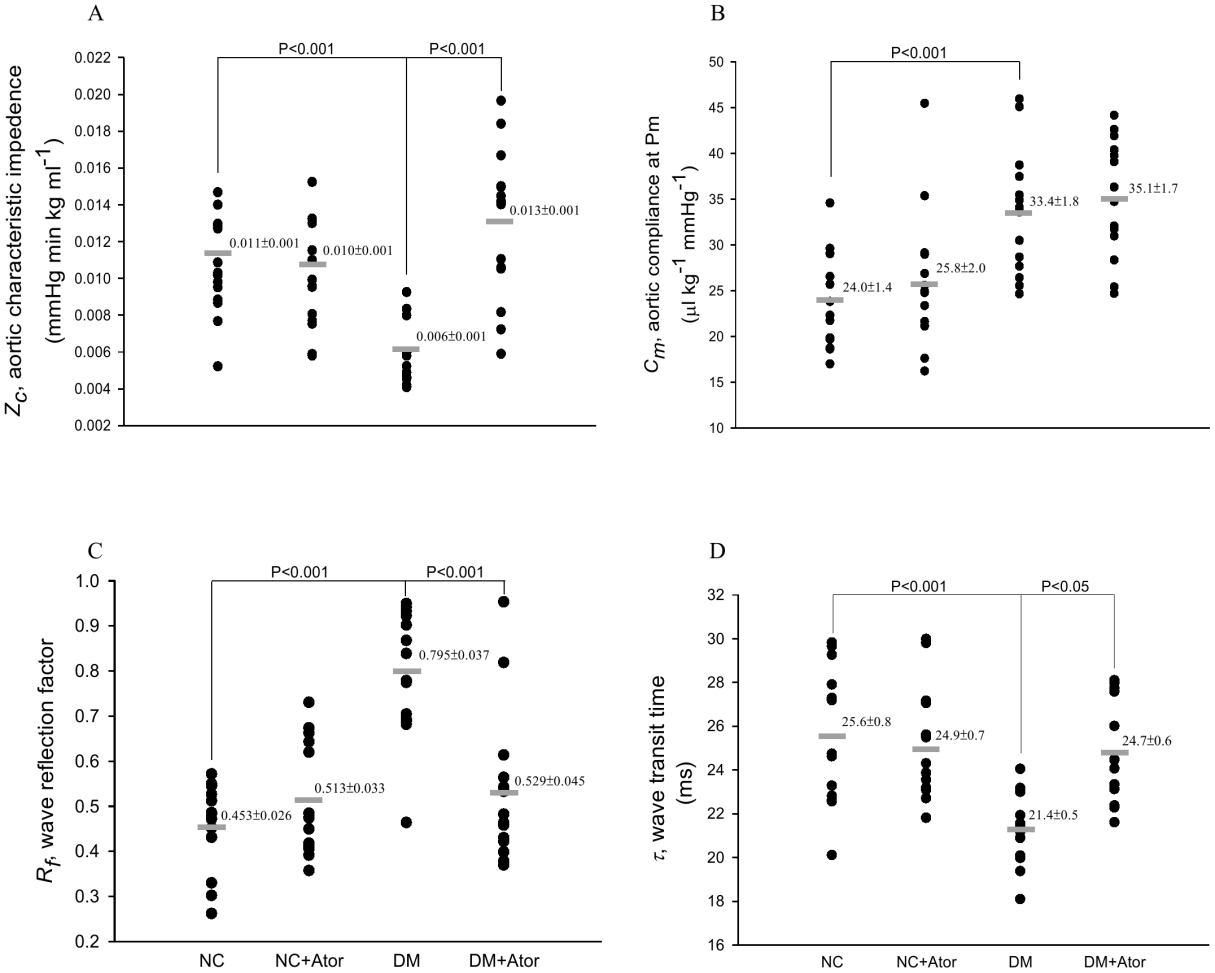
Effects of diabetes and Ator on the oscillatory hemodynamic loads such as aortic characteristic impedance (*Z*_c_ in A), systemic arterial compliance at mean aortic pressure (*C_m_* in B), wave reflection factor (*R_f_* in C), and wave transit time (τ in D). Data are expressed as means ± s.e. NC, normal controls; DM, STZ-diabetic rats; Ator, atorvastatin; STZ, streptozotocin. (*n* = 14 in each group).

Figure 4 demonstrates the effects of diabetes and Ator on the pulsatile nature of blood flows in arteries in terms of aortic characteristic impedance (*Z_c_* in A), aortic compliance (*C_m_* in B), wave reflection factor (*R_f_* in C), and wave transit time (τ in D). The diabetes-derived fall in *Z_c_* was prevented by administration of Ator to the DM. However, for rats treated with Ator, no change was observed for the diabetes-induced rise in *C_m_*. By contrast, Ator therapy attenuated the diabetes-related deterioration in pulse wave reflection, as evidenced by both the increase of 15.4% in τ (*P*<0.05), and the decrease of 33.5% in *R_f_* (*P*<0.001). By contrast, the oscillatory components of the ventricular afterload, including *Z_c_*, *C_m_*, τ and *R_f_*, were not modified by administration of Ator to the NC.

**Figure 5:**
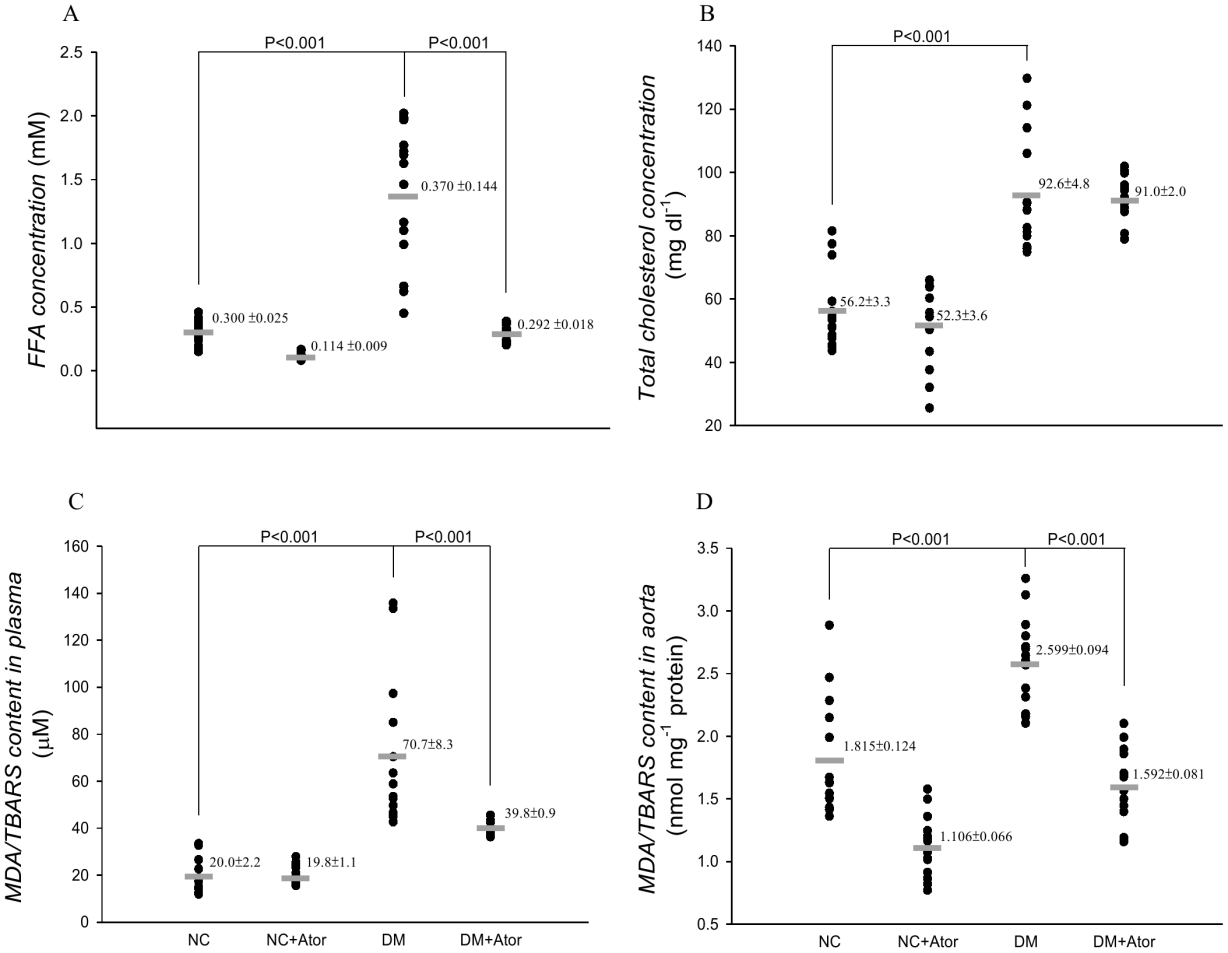
Effects of diabetes and Ator on plasma levels of FFA (A) and total cholesterol (B) and plasma (C) and aorta levels (D) of MDA. Data are expressed as means ± s.e. NC, normal controls; DM, STZ-diabetic rats; Ator, atorvastatin; STZ, streptozotocin; MDA, malondialdehyde. (*n* = 14 in each group).

Compared with the NC, the DM exhibited elevated plasma levels of FFA (Fig. 5A), and total cholesterol (Fig. 5B), and plasma (Fig. 5C), and aorta levels (Fig. 5D) of MDA. The high plasma levels of total cholesterol in the DM did not change in response to the low-dose treatment with Ator. By contrast, the diabetes-related increase in plasma levels of FFA was attenuated by the administration of Ator to the DM. The Ator therapy also ameliorated the diabetes-induced deterioration in lipid peroxidative status, as evidenced by the reduction of 43.7% in plasma (*P*<0.001) and of 38.7% in aorta levels of MDA (*P*<0.001). Conversely, Ator therapy exerted no effects on those biochemical and metabolic data in the NC.

**Figure 6:**
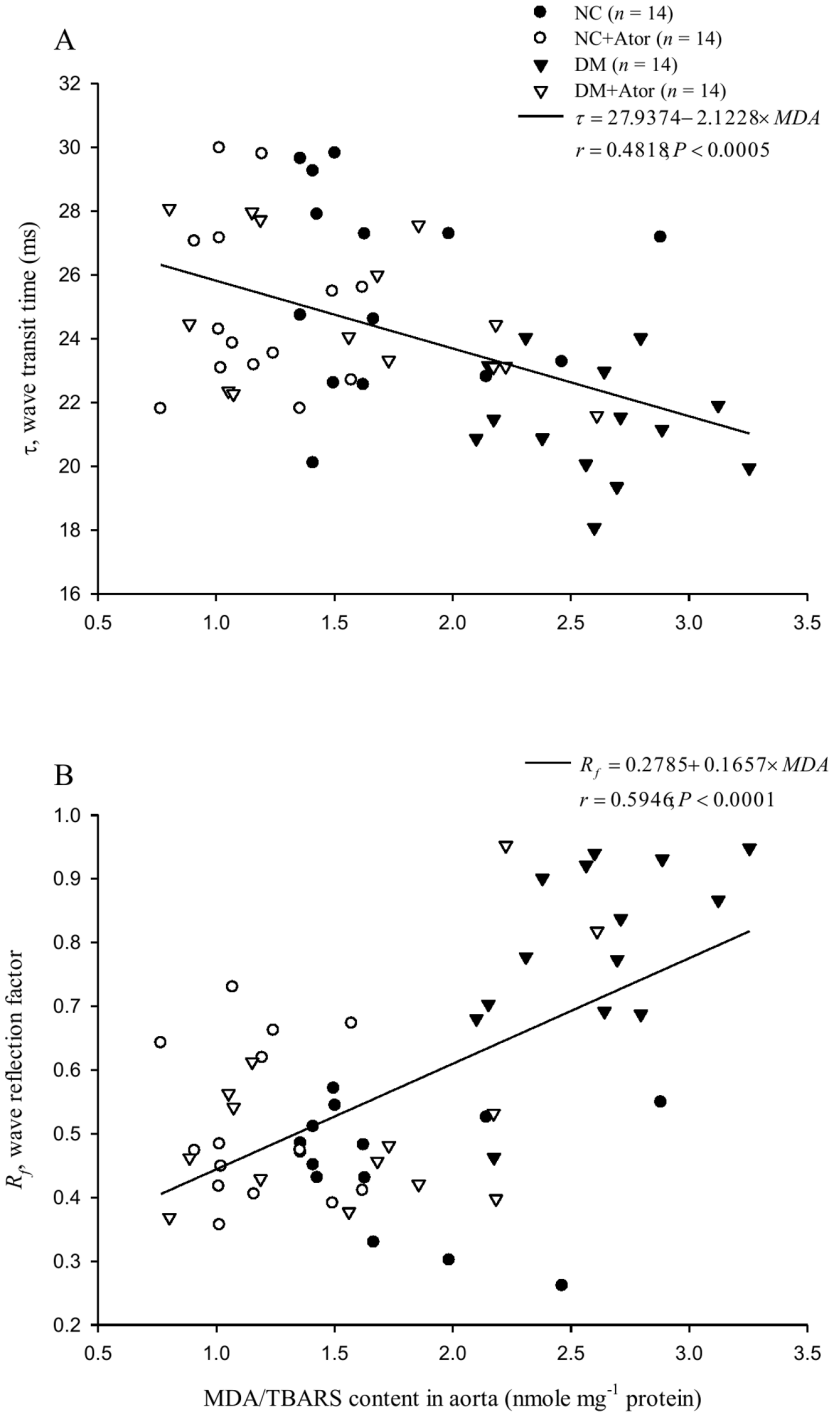
In A, noted was the significant inverse linear correlation between the arterial wave transit time (τ) and aorta levels of MDA in the diabetic rats administered Ator. In B, the correlation between the arterial wave reflection factor (*R_f_*) and aortic MDA content reached significance. Ator, atorvastatin; MDA, malondialdehyde.

Figure 6A shows the relationship between arterial τ and aorta levels of MDA in the diabetic rats treated with Ator. The significant inverse linear correlation between arterial τ and aortic MDA content was noted (τ = 27.9374–2.1228× MDA; *r* = 0.4818, *P*<0.0005). Figure 6B shows the prediction of arterial *R_f_* from the aorta levels of MDA in the diabetic animals under Ator. The correlation between these two parameters reached significance (*R_f_* = 0.2785 + 0.1657× MDA; *r* = 0.5946, *P*<0.0001).

**Figure 7:**
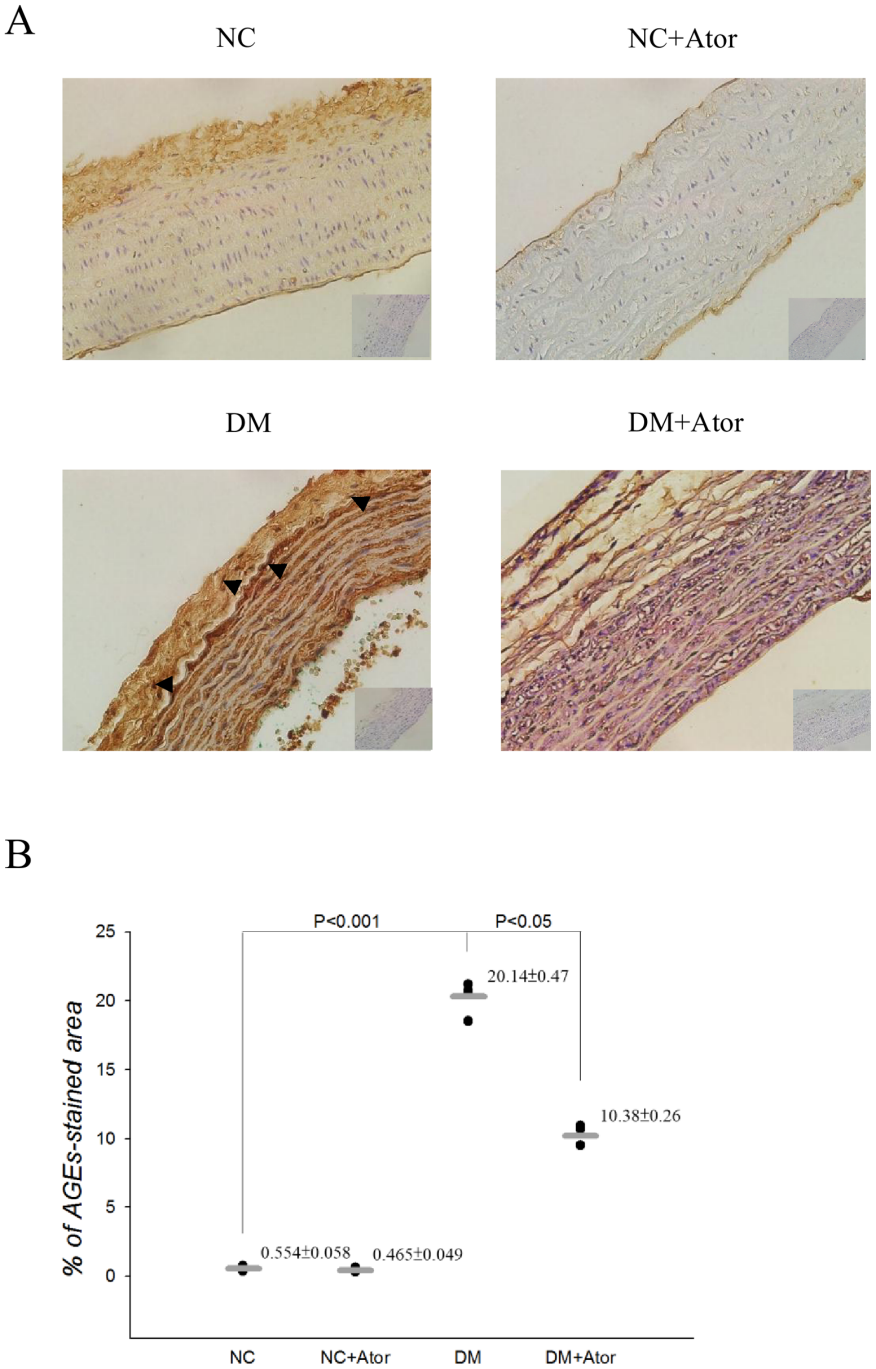
Effects of diabetes and Ator on the expression of aortic AGEs (A) and their quantities (B). AGE expressions were probed using immunohistochemical staining (400x). The arrows indicated the sites of antibody staining. ImageScope Viewer^R^ was used to calculate the percentage of AGE staining: positivity (%)  =  total number of positive pixels divided by total number of pixels. Data are expressed as means ± s.e. NC, normal controls; DM, STZ-diabetic rats; AGEs, advanced glycation end products; Ator, atorvastatin. (*n* = 3 in each group).

**Figure 8:**
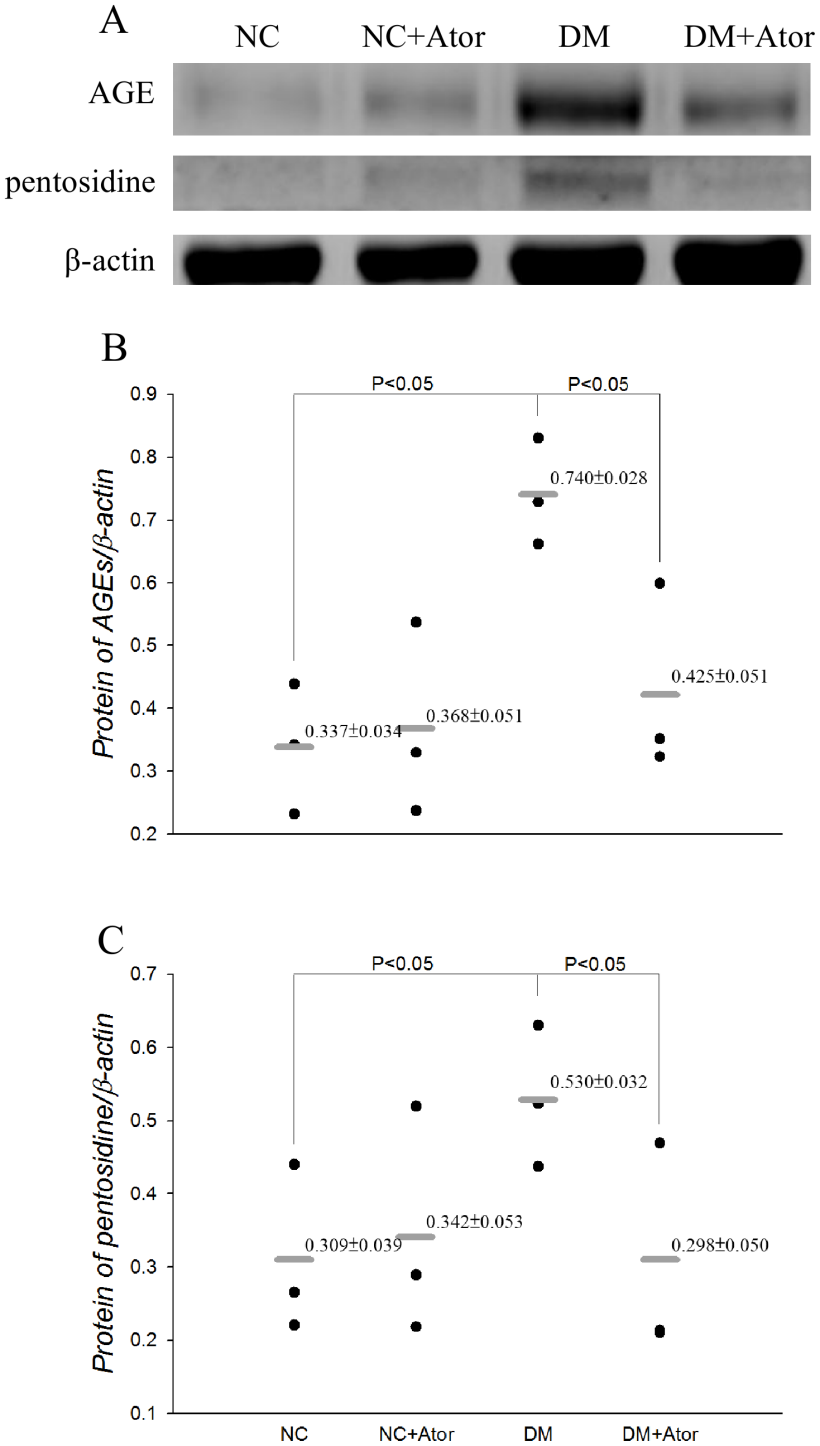
Effects of diabetes and Ator on aortic AGEs and pentosidine measured by western blotting technique. Protein expression was normalized to β-actin. Data are expressed as mean ± s.e. NC, normal controls; DM, STZ-diabetic rats; AGEs, advanced glycation end products; Ator, atorvastatin. (*n* = 3 in each group).

Figure 7 shows the effects of diabetes and Ator on the expression of AGEs (A) and the quantification of immunohistochemical staining (B) in the aortas. The Ator therapy attenuated the diabetes-induced deterioration in tissue collagen-crosslinks, as evidenced by the reduction of 48.5% in aorta levels of AGEs (*P*<0.05). The results were congruent with those obtained by western blotting technique, which were shown in Fig. 8. Also observed was the significant difference in aorta levels of pentosidine between the Ator-treated DM and the untreated diabetic controls (Fig. 8C).

## Discussion

Our significant findings were that without affecting the plasma levels of total cholesterol, the low-dose treatment with Ator might protect the diabetic vasculature against diabetes-associated deterioration in arterial wave properties and aorta stiffness. The cholesterol-independent effects of Ator on vascular dynamics were in parallel with its lowering of lipid oxidation-derived MDA contents in plasma and aortic walls in diabetes.

The DM exhibited isobaric vasodilatation, an increase in blood flow that occurred in the absence of any significant change in arterial blood pressure, resulting in a decrease in *R_p_* (Fig. 3D). It has been shown that increased FFAs can act in concert with high blood sugar to enhance superoxide production impairing the contractile status of the vascular smooth muscle cells (VSMCs) [3], [29]. Furthermore, excessive MDA adducts to lysine are reported to promote vascular complication through induction of inflammatory pathways [30]. Thus, the contractile dysfunction of VSMCs by those molecular events may lengthen the diabetic resistance vessels, causing an increase in arteriolar diameter and thus a fall in *R_p_* [31]. Although Ator, by lowering the plasma FFA levels, could act as a preventive agent in diabetic vascular complications, no beneficial effects of Ator on resistance to blood flows in arteries were observed in this experimental diabetes. The ability of Ator to lower MDA content also had no benefits for the physical properties of the resistance vessels in the DM.

To aortic distensibility of the DM, the physiological implication of reduced *Z_c_* (Fig. 4A) seems to conflict with that of diminished τ (Fig. 4D). Decreased *Z_c_* may be indicative of more distensible aortas, whereas diminished τ may have an opposite physical meaning [16], [18]. With unaltered aortic pressure, a decline in *Z_c_* suggests that the contractile function of the VSMCs may be impaired in Windkessel vessel [29]. Under isobaric vasodilatation, the inactivation of the VSMCs has the potential to elevate the elastic modulus of the aortic wall and cause a fall in aortic distensibility [16]. Meanwhile, the contractile dysfunction of the diabetic aortas probably lengthens the VSMCs, resulting in an increase in aortic lumen diameter that can cause a fall in *Z_c_*. Because the net results of diabetes on *Z_c_* would depend on the relative influence of those counter-balancing factors, i.e., pulse wave velocity and aortic cross-sectional area, there is difficulty using *Z_c_* to describe the aortic distensibility in the DM. By contrast, being relatively independent to body shape, wave transit time (τ), which is inversely related to pulse wave velocity, could be used to describe the aortic distensibility: the stiffer the aortic wall, the shorter the wave transit time and vice versa [16]. Thus, a decline in aortic distensibility in the DM could be reflected in the reduction in wave transmission time along the path.

The development of increasing collagen-crosslinks by AGEs has been proposed as one of the major contributing factors responsible for arterial stiffening [32]. In this study, the low-dose treatment with Ator attenuated the diabetes-related fall in aortic distensibility, as evidenced by the significant increase in τ (Fig. 4D). The prevention of diabetes-related aorta stiffness by Ator was in parallel with the reduction in aorta levels of AGEs and pentosidine (Fig. 7 or 8). This finding was in accordance with the report from Jinnouchi et al. [33] that Ator therapy could decrease serum AGE levels via its anti-oxidative property in diabetes. We also found that there existed a significant inverse linear correlation between the arterial τ and the aortic MDA content (Fig. 6A). Because MDA can react with collagen to form MDA-collagen cross-links [8], [9], the Ator therapy might protect against arterial stiffening, possibly through its reduction of MDA content in the diabetic aortas (Fig. 6A). High concentrations of FFAs in diabetes also changed the extracellular matrix produced by the VSMCs [34], increasing vascular smooth muscle tone and further diminishing arterial distensibility. The decreased plasma levels of FFAs by Ator (Fig. 5A) might be another crucial factor attenuating aorta stiffness in the DM.

Changes in arterial wave properties are considered critical determinants of the cardiac muscle cells to adapt to hypertrophy [18]. In this study, a reduction in τ suggested that diabetes caused an early return of the pulse wave reflection from the peripheral circulation. The treatment of the DM with Ator retarded this early return of the pulse wave reflection (Fig. 4D). The diabetic syndrome also contributed to a significant rise in *R_f_* (Fig. 4C), indicating that the heavy reflection intensity occurred in the DM. Treating the DM with Ator resulted in a significant fall in *R_f_*, suggesting that the heavy reflection phenomenon was alleviated. The increased τ associated with the decreased *R_f_* indicated that Ator, by diminishing the MDA content in diabetes (Fig. 6), improved the systolic loading conditions for the LV coupled to its vasculature. The decline in systolic load by Ator could be responsible for the prevention of diabetes-related cardiac hypertrophy, as evidenced by the reduction of LVW-to-BW ratio (Table 1).

Just as the elastic modulus is an expression used to characterize the material properties, distensibility is a term used to describe the elastic behavior of a hollow vessel. Compliance and distensibility differ because compliance is equal to distensibility times volume [35]. In this study, the DM indicated an increase in aortic compliance at *P_m_* (Fig. 4B). The decreased distensibility associated with the increased compliance suggested that volume expansion in the vasculature might exist in the DM. The volume expansion in diabetes is supported by other reports in the literature [36], [37]. The low-dose Ator administered to the DM for 6 weeks produced no significant alteration in *C_m_*. Therefore, the diabetes-related abnormality in volume expansion could be prevented using Ator therapy, as manifested by the increased distensibility associated with the unaltered *C*_m_.

Certain limitations of this study deserve consideration. Because aortic input impedance cannot be measured in conscious animals, an evaluation of the effects of pentobarbital anesthesia on rats is impossible. The results reported here pertain only to measurements made in anesthetized rats in open-chest conditions. This condition might induce changes in aortic pressure profiles and introduce reflex effects not observed in closed-chest conditions. The degree to which anesthesia and thoracotomy influence pulsatile hemodynamics in rats is uncertain. However, studies using other animal models have suggested that the effects are small, relative to the biological and experimental variability between animals [38].

Overall, the STZ-diabetic rats showed a detriment to the pulse wave reflection phenomena in relation to τ and *R_f_*. After a low-dose treatment with Ator, an increase in τ suggested that the positive pleiotropic effects of the drug may prevent the diabetes-induced decline in aortic distensibility, which paralleled its reduction of MDA contents in elastic arteries. The increased τ associated with the decreased *R_f_* indicated that Ator could attenuate the diabetes-related augmentation in systolic loading conditions for the left ventricle, preventing cardiac cells from hypertrophy. Based on these results, we suggest that the low-dose Ator therapy ameliorates the vascular complications observed in the DM, at least partly through its ability to reduce the plasma and aorta levels of lipid oxidation-derived MDA.
